# The larva of *Adicella syriaca* Ulmer 1907, including a key to the European larvae of *Adicella* McLachlan, 1877 (Trichoptera, Leptoceridae)

**DOI:** 10.3897/zookeys.711.20121

**Published:** 2017-10-23

**Authors:** Johann Waringer, Hans Malicky, Wolfram Graf, Simon Vitecek

**Affiliations:** 1 Department of Limnology and Bio-Oceanography, University of Vienna, Althanstrasse 14, A-1090 Vienna, Austria; 2 Sonnengasse 13, A- 3293 Lunz am See, Austria; 3 Institute of Hydrobiology and Aquatic Ecology Management, University of Natural Resources, Gregor Mendelstr. 33, A-1180 Vienna, Austria; 4 Senckenberg Research Institute and Natural History Museum, Senckenberganlage 25, 60325 Frankfurt am Main, Germany

**Keywords:** Description, distribution, larva, identification, West Palearctic fauna

## Abstract

*Adicella syriaca* is a leptocerid caddisfly distributed throughout the Balkan Peninsula, the Carpathians, the Hungarian Lowlands, the Pontic Province, and the Caucasus. This paper describes the previously unknown larva of this species, based on material from the Greek island of Corfu. Information on the morphology of the fifth larval instar is given, and the most important diagnostic features are illustrated. A key to the known larvae of the European species of *Adicella* McLachlan, 1877 is provided. In the context of existing identification keys, the larva of *Adicella syriaca* Ulmer, 1907 keys together with *Adicella cremisa* Malicky, 1972, but the species pair can be easily separated by the number of setae on the pro- and mesonotum, and setation patterns on abdominal dorsum IX.

## Introduction

Eleven species of *Adicella* McLachlan, 1877 are currently known in Europe (Graf et al. 2008; Malicky 2004, 2005a). However, with respect to larval taxonomy, descriptions for only four species were uncovered: *Adicella meridionalis* Morton, 1906 (Vieira-Lanero et al. 1997, Vieira-Lanero 2000), *A. filicornis* (Pictet, 1834), *A. reducta* (McLachlan, 1865) (Wallace et al. 2003, Waringer and Graf 2011) and *A. cremisa* Malicky, 1972 (Graf et al., submitted). However, of the remaining seven species where larvae are unknown, Malicky collected larvae of *A. syriaca* on the Greek island of Corfu. *Adicella syriaca* was described by Ulmer, 1907, based on material from Lebanon (Morse 2017); the species is rather widely distributed throughout Europe, ranging from the Balkans through the Carpathians and Hungarian Lowlands to the Caucasus (Ćukušić et al. 2017; Graf et al. 2008; Ibrahimi et al. 2012; Morse 2017; Živić et al. 2006). With our description of its larva and the key, proposed here, the identification of five out of eleven European *Adicella* species is now possible, without an adult male specimen as frequently required in caddisfly studies.

## Materials and methods

Two final instar larvae and many adults of *Adicella syriaca* were collected by Malicky at Mesaria on the island of Corfu, Greece (39°44'N, 19°44'E, 40 m a.s.l.) on 1 May 1979. Larval caddisflies were picked from the mineral substrate with forceps, and adults were collected using light traps. The material was preserved in 70% ethanol. The larvae were studied and photographed using a Nikon SMZ 1500 binocular microscope with DS-Fi1 camera and NIS-elements D 3.1 image stacking software for combining 8–45 frames in one focused image. Larval morphological features are named following Wiggins (1998) and Waringer and Graf (2011), nomenclature of primary setae and setal areas (= sa) follows Wiggins (1998). Species association was enabled by the fact that final instar larvae and adults were collected at the same location; in addition, the other four Leptoceridae species known from Corfu are well known in the larval stage (*Leptocerus interruptus* (Fabricius, 1775), *L. tineiformis* Curtis, 1834, *Mystacides azurea* (Linnaeus, 1761): Wallace et al. 2003; Waringer and Graf 2011); *Triaenodes ochreellus lefkas* Malicky, 1974: Corallini Sorcetti and Moretti 1984; Vieira-Lanero (2000)). Although the location was repeatedly sampled, *A. syriaca* was the only *Adicella* species on this island. Final instar larvae and adults of *Adicella syriaca* used for the descriptions are deposited in the collection of Hans Malicky (Lunz am See, Austria). Comparative larval material of *Adicella cremisa*, *A. filicornis* and *A. reducta* is deposited in the collections of W. Graf and J. Waringer (Vienna, Austria). The larval material is intended to be subsequently transferred to Austrian Museum collections.

## Results

### Description of the fifth instar larva of *Adicella syriaca*

#### Adicella syriaca

Taxon classificationAnimaliaTrichopteraLeptoceridae

Ulmer, 1907

##### Diagnosis.

Head with pattern composed of dark bands and dark muscle attachment spots; case with spiral pattern, constructed of plant material; metanotal sa3 reduced to a single seta per side; pronotum with 56–65 setae of varying length per pronotal half; total number of setae per mesonotal sclerite 11–13; outermost seta of abdominal dorsum IX setal group approximately as long as width of this segment.

##### Biometry.

Body length ranging from 6.8 to 7.7 mm, head width from 0.58 to 0.60 mm (n = 2).

##### Head.

**Figures 1–6.**
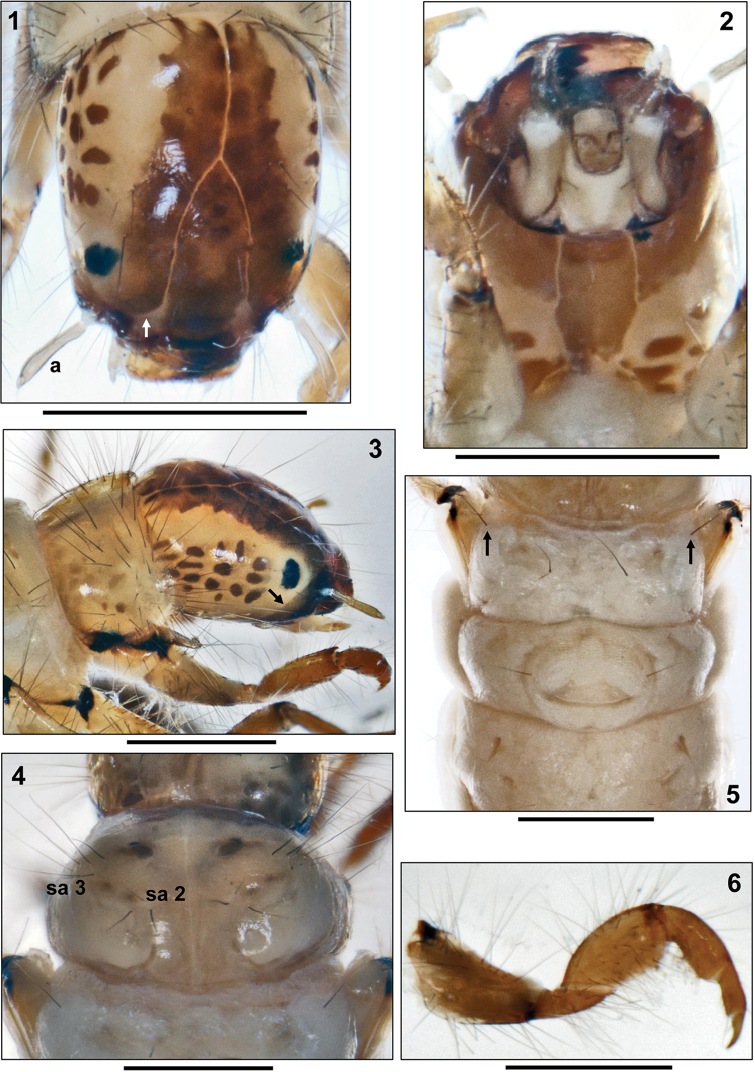
*Adicella syriaca* Ulmer 1907, final instar larva. **1** Head, dorsal view (a= antenna; arrow= subocular ecdysial line) **2** Head, ventral view **3** Head and pronotum, right lateral view (arrow= subocular ecdysial line) **4** Mesonotum, dorsal view (sa2, sa3= setal areas 2 and 3) **5** Metathorax and abdominal segments I and II, dorsal (arrows = single seta of sa3) **6** Right foreleg, posterior face. Scale bars: 0.5 mm.

Head capsule surface smooth, with very shallow wrinkles, elongated and hypognathous. Base coloration pale yellow, with dark, reddish brown, oval muscle attachment spots on lateral and postero-ventral sections of parietalia. Frontoclypeus and parietal bands along frontoclypeal and coronal sutures dark reddish brown (Figs 1–3). White ring present around eyes (Fig. 3). Complete set of primary setae present (Figs 1–3). Frontoclypeus elongated, narrow, without central constriction (Fig. 1). Subocular ecdysial line running from foramen occipitale to ventro-lateral section of parietalia. Anteriorly of the eyes the subocular ecdysial line bends dorsally, eventually meeting frontoclypeal suture in a straight line (Fig. 3, arrow). Antennae slender, approximately six times longer than their basal width, situated at extreme anterior end of parietalia and originating from a socket-like ridge; antennal apex with single seta (Fig. 1a). Labrum light brown, quadrangular, with anterior median notch, ventral brush and six pairs of primary setae (Fig. 1). Ventral apotome medium brown, with darker brown anterior border, elongated quadrangular, with irregular lateral and posterior sides (Fig. 2). Mandibles black, each with ventral and dorsal cutting edge and terminal teeth along edge (Fig. 2).

##### Thorax.

Pronotum yellowish brown, with dark brown roundish muscle attachment spots (Fig. 3); with continuous row of widely-spaced, straight, black setae along anterior border; pronotal surface densely covered by 56–65 black setae of varying length per pronotal half (Fig. 3). Pleural sclerites irregular, elongated, pale, with black ventral margins; anteriorly, with brownish, finger-like protrochantin with blunt tip bearing one dark terminal seta (Fig. 3). Prosternal horn absent.

Mesonotum covered by two sclerites, each posterior half with large, semicircular central constriction; sclerites pale yellow, with distinct markings and muscle attachment spots along anterior margin and at center (Fig. 4). Total number of setae of varying lengths per mesonotal sclerite is 11–13 (sa1 without setae, each sa2 with 3 setae, each sa3 with 8–10 setae; Fig. 4). Mesopleurites pale, with narrow, blackish central bar (Fig. 3). Mesoventer without setae.

Metanotum without sclerotization except pleural sclerites; metanotal sa1 without setae, each sa2 with 1 seta each, sa3 reduced to a single seta per side (Fig. 5, arrows). Metaventer with a row of 4–5 setae per side (Fig. 11). Pleural sclerite arrangement as on mesonotum.

**Figures 7–12.**
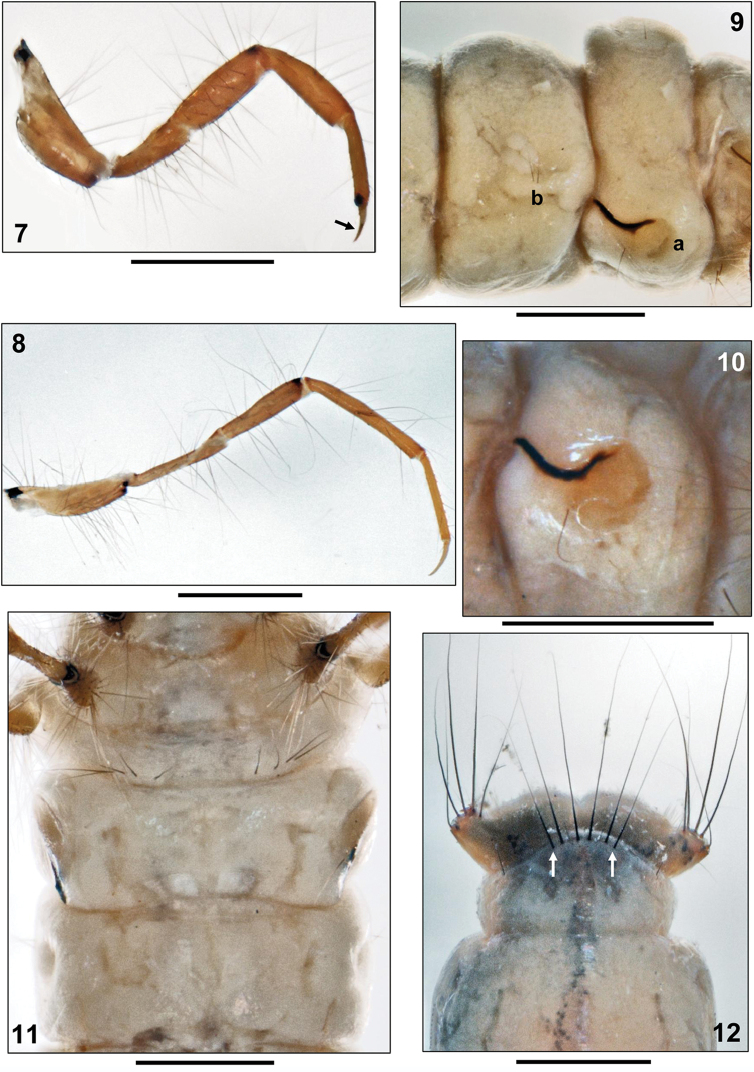
*Adicella syriaca* Ulmer 1907, final instar larva. **7** Right midleg, posterior face (arrow: tarsal claw not hook-shaped) **8** Right hind leg, posterior face **9** Abdominal segments I and II, right lateral (a= lateral sclerite; b= lateral setae) **10** Lateral sclerite, detail **11** Sterna of metathorax and abdominal segments I and II **12** Tip of abdomen, dorsal (arrows= outermost setae of ninth abdominal tergite). Scale bars: 0.5 mm.

Legs orange-yellow, with very numerous setae, especially on coxae, trochanters, and femora (Figs 6–8); tibiae and tarsi undivided and without central constrictions. Femur of foreleg much wider than those of mid- and hind legs. Claw of mid leg curved and not hook-shaped as in genus *Leptocerus* (Fig. 7, arrow). Long fringes of swimming setae absent on hind legs.

##### Abdomen.

**Figures 13–19.**
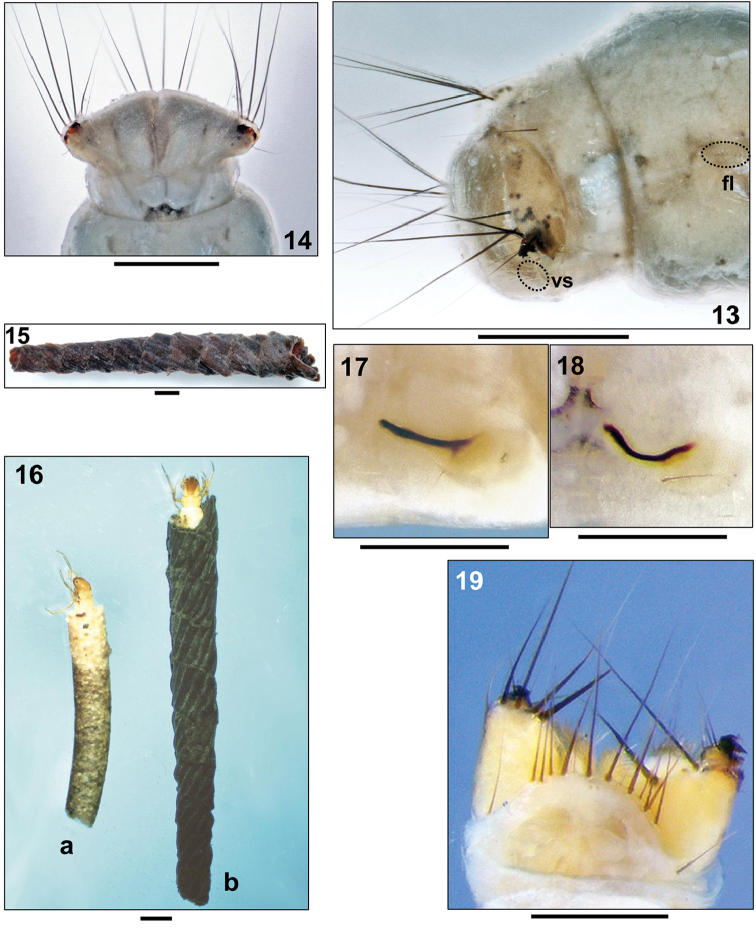
**13–15** *Adicella syriaca* Ulmer, 1907, final instar larva: **13** Tip of abdomen, right lateral (fl= forked lamellae on segment VIII; vs= ventral setae on segment IX) **14** Tip of abdomen, ventral **15** Larval case, right lateral **16** Final instar larvae in their cases. **a** *Adicella filicornis* (Pictet, 1834) **b** *Adicella reducta* (McLachlan, 1865) **17–18** Lateral sclerites on abdominal segment I of fifth instar larvae, right lateral view: **17** *Adicella reducta* (McLachlan, 1865) **18** *Adicella cremisa* Malicky, 1972 **19** *Adicella filicornis* (Pictet, 1834), final instar larva. Tip of abdomen, dorsal. Scale bars: 0.5 mm (except Figs 15, 17: 1 mm).

Abdomen white, cylindrical. First abdominal segment with one dorsal and two lateral protuberances (Fig. 9); dorsal sa1 and sa3 not developed, dorsal sa2 with single seta on each side (Fig. 5); oval and light orange lateral sclerite with strongly sclerotized, dark, curved and sickle-shaped posterior process; lateral sclerite with 1 ventral seta (Figs 9, 10). Abdominal tergum IX with pale, weakly sclerotized tergite, bearing 6 long and 4 short terminal setae; abdominal segment IX with 1 posterodorsal seta on either side (Fig. 12). Outermost seta on abdominal dorsum IX approximately as long as width of segment IX (Fig. 12, arrows). Anal prolegs pale and weakly sclerotized, each with large lateral sclerite and more strongly sclerotized anal claw with two tiny accessory hooks (Fig. 13). Each lateral sclerite bearing several long, black setae (Figs 13, 14). Each anal proleg medially with small group of pale, soft ventral setae (Fig. 13vs); tooth-edged plates around anal slit absent (Fig. 14). Gills and lateral line not visible; however, a lateral row of forked lamellae is present on abdominal segment VIII (Fig. 13fl).

##### Case.

In the final instar larvae, straight, cylindrical, tapering, constructed of equally sized pieces of thin plant stems and roots arranged in a typical single spiral (Fig. 15). Case length 12.2–13.9 mm, anterior width 1.9–2.2 mm, posterior width 1.0–1.2 mm (n= 2).

### Morphological separation of fifth instar larvae of *Adicella syriaca* from other European species of Leptoceridae and *Adicella*

A summary of morphological features for the identification of Leptoceridae larvae was provided by Wallace et al. (2003) and of Triaenodini larvae by Morse (1981). Within the framework of available leptocerid keys by Waringer and Graf (2011) and Graf et al. (2017), and the descriptions of Vieira-Lanero et al. (1997), Vieira-Lanero (2000), and Graf et al. (2017), *A. syriaca* is characterised by the following features:

– head with pattern composed of dark bands and dark muscle attachment spots (Figs 1, 3);

– metanotal sa3 reduced to a single seta per side (Fig. 5, arrows);

– pronotum with 56–65 setae of varying length per pronotal half (Fig. 3);

– total number of setae per mesonotal sclerite 11–13 (Fig. 4);

– lateral sclerites on 1^st^ abdominal segment each with dark stripe, bent (Figs 10, 18) and not straight (Fig. 17);

– outermost seta of abdominal dorsum IX setal group (Fig. 12, arrow) approximately as long as width of this segment.

### Key to the known final instar *Adicella* larvae of Europe

**Table:** 

1	Head uniformly orange, without pattern; case cylindrical, smooth, constructed of mineral particles (Fig. 16a)	**2**
–	Head pale, with pattern composed of dark bands and dark muscle attachment spots; case with spiral pattern, constructed of plant material (Figs 1, 16b)	**3**
2	Abdominal dorsum IX (including both posterolateral setae) with 12 setae; species endemic to Iberic-Macaronesian Region (European Ecoregion I)	***Adicella meridionalis* Morton, 1906**
–	Abdominal dorsum IX (including both posterolateral setae) with 14–15 setae (Fig. 19); species widespread outside of European Ecoregion I	***Adicella filicornis* (Pictet, 1834)**
3	Metanotal sa3 with 13–18 setae per side (Fig. 20)	***Adicella reducta* (McLachlan, 1865)**
–	Metanotal sa3 reduced to a single seta per side (Fig. 5, arrows)	**4**
4	Pronotum with 56–65 setae of varying length per pronotal half (Fig. 3); total number of setae per mesonotal sclerite is 11–13 (Fig. 4); outermost seta of abdominal dorsum IX setal group (Fig. 12, arrow) approximately as long as width of this segment	***Adicella syriaca* Ulmer, 1907**
–	Pronotum with 35–37 setae of varying length per pronotal half; total number of setae per mesonotal sclerite is 7–8; outermost seta on abdominal dorsum IX approximately half as long as width of this segment (Fig. 21)	***Adicella cremisa* Malicky, 1972**

**Figure 20–21.**
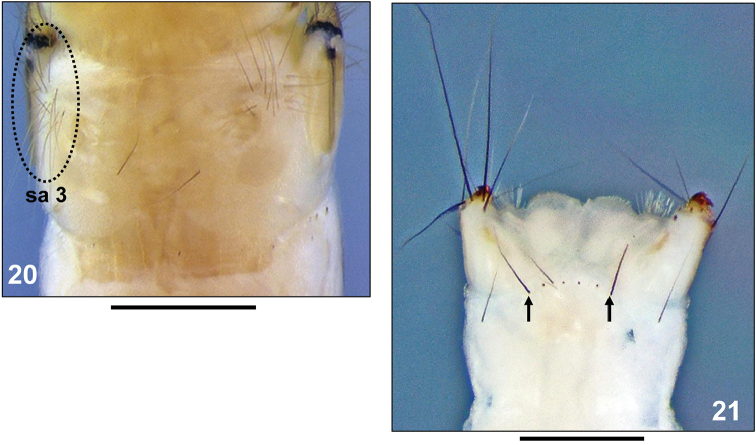
**20** *Adicella reducta* (McLachlan, 1865), final instar larva. Metanotum, dorsal view (sa3 = setal area 3) **21** *Adicella cremisa* Malicky, 1972, final instar larva. Tip of abdomen, dorsal (four setae are missing and only their alveolae visible). Scale bars: 0.5 mm.

## Discussion

The larvae of *Adicella* species frequent a large range of habitats, including small shallow springs, rocky streams, marshes, canals and rivers, and often colonize root mats of riparian vegetation, with *A. reducta* remaining the only leptocerid caddisfly to persevere in large impoverished streams (Wallace et al. 2003). Cianficconi and Moretti (1987) also collected larvae of *A. cremisa* in standing water bodies and irrigated meadows. On Corfu, *A. syriaca* is most common relatively close to the shore (Malicky 2005b) where it inhabits streams and rivulets, and also mill brooks, shaded by *Nereum oleander*, *Arundo donax*, *Platanus orientalis*, *Ficus carnica*, and *Inula viscosa*. According to mandible morphology, *Adicella* larvae are shredders and, to a minor extent, also grazers (Graf et al. 2008); this also fully applies to *A. syriaca* where mandibles are fitted with ventral and dorsal cutting edges and terminal teeth along edges (Fig. 2).

The distribution of *Adicella syriaca* ranges from Tunisia, the Levant, and Turkey, throughout the Balkan Peninsula to Hungary and the Caucasus (Graf et al. 2008; Malicky, 2004, 2005a, b, 2014; Morse 2017). In Greece, *A. syriaca* is widespread on the mainland, but also on many islands, e.g., Euboea, Corfu, Lefkada, Kefallonia, Samothraki, Skiathos, Samos, Lesbos, Andros, and Rhodes (Malicky, 2005b).

The collection time of final instar larvae of *A. syriaca* in May fits well into the reported flight period of adults from the onset of April to mid-November. Within this period, a peak in May-June and in October can be observed which might be an indication of two generation cycles per year (Malicky 2005b). In *A. cremisa*, Graf et al. (2017) observed adults flying amongst dense riparian vegetation in vertical zig-zag patterns of about 10 cm extent; the long whitish antennae obviously played a role as an optical cue in courtship behavior in this species.

## Supplementary Material

XML Treatment for Adicella syriaca
